# Modulating the Biliverdin Reductase (BVR)/ERK1/2 Axis to Attenuate Oxidative Stress in Rat Arterial Rings

**DOI:** 10.5812/ijpr-156828

**Published:** 2024-12-04

**Authors:** Kuldeepak Sharma, Mateja Skufca Sterle, Hugon Mozina

**Affiliations:** 1 Department of Medicine, Institute of Pharmacology and Toxicology, University of Ljubljana, Ljubljana, Slovenia; 2 Prehospital Unit, Community Health Centre, Ljubljana, Slovenia; 3 Internal Medicine First Aid, University Clinical Center, Ljubljana, Slovenia

**Keywords:** Reactive Oxygen Species, Biliverdin Reductase, Biliverdin, Bilirubin

## Abstract

**Background:**

Biliverdin reductase (BVR) plays a central role in bile pigment metabolism by reducing biliverdin (BV) to bilirubin (BR), a potent antioxidant that scavenges reactive oxygen species (ROS) under normal and pathological conditions. Elevated oxidative stress activates extracellular signal-regulated protein kinases 1/2 (ERK1/2) signaling, which strongly interacts with BVR’s C and D motifs, forming the BVR/ERK1/2 axis. In pathological states, increased ERK1/2 activity inhibits BVR’s ability to convert BV to BR, exacerbating oxidative damage and contributing to cardiovascular disease. Therefore, the interaction between BVR and ERK1/2 is critical in modulating oxidative stress.

**Objectives:**

This study aimed to evaluate the effects of BR and the ERK1/2 inhibitor PD-98059, both individually and in combination, on ROS levels, ERK1/2 activity, and vascular responses under normoxic and hypoxia-reoxygenation (H-R) injury conditions.

**Methods:**

Aortic rings from rats were subjected to equal distending pressure after oxidative stress induction using 22'-Azobis (2-amidinopropane) dihydrochloride (ABAP) in an organ bath. Different doses of BR were administered in combination with the ERK1/2 inhibitor PD-98059 to assess their impact on ROS depletion, vascular relaxation, and maximal effect (Emax).

**Results:**

The combination of BR and PD-98059 significantly enhanced aortic relaxation and Emax under both normoxic and H/R conditions compared to either treatment alone. Inhibiting ERK1/2 with PD-98059 appeared to upregulate BVR activity, increasing BR synthesis and reducing oxidative damage in aortic rings.

**Conclusions:**

Biliverdin reductase plays a vital role in defending against oxidative stress and endothelial dysfunction through its dual-specificity kinase activity and interaction with ERK1/2. ERK1/2 inhibition further enhances BR’s ROS-scavenging ability and vascular protective effects. Targeting the interaction between BVR and ERK1/2 holds potential as an effective therapeutic strategy for conditions characterized by excessive ROS levels, such as cardiovascular diseases.

## 1. Background

An imbalance between the activity of endogenous pro-oxidative enzymes (such as xanthine oxidase, nicotinamide adenine dinucleotide phosphate (NADPH) oxidase, or the mitochondrial respiratory chain) and anti-oxidative enzymes (e.g., catalase, paraoxonase, glutathione peroxidase, heme oxygenase, biliverdin reductase (BVR), thioredoxin peroxidase/peroxiredoxin, and superoxide dismutase) during oxidative stress is the primary trigger for reactive oxygen species (ROS) production (1, 2). Elevated ROS levels reduce the availability of functional nitric oxide (NO), leading to the generation of detrimental peroxynitrite. Peroxynitrite, in turn, has the ability to "uncouple" endothelial NO synthase, converting it into a dysfunctional superoxide-producing enzyme that exacerbates vascular oxidative stress (3). Thus, the accumulation of ROS is a major factor contributing to the increasing prevalence of heart failure, hypertension, congenital heart failure, vascular diseases, coronary artery disease, and stroke, particularly with aging (4). Additionally, key risk factors for ROS include obesity, diabetes, cigarette smoking, unhealthy and sedentary lifestyles, and genetic susceptibility (5). Numerous animal models have further confirmed the role of ROS in cardiovascular dysfunction (6).

**Figure 1.**
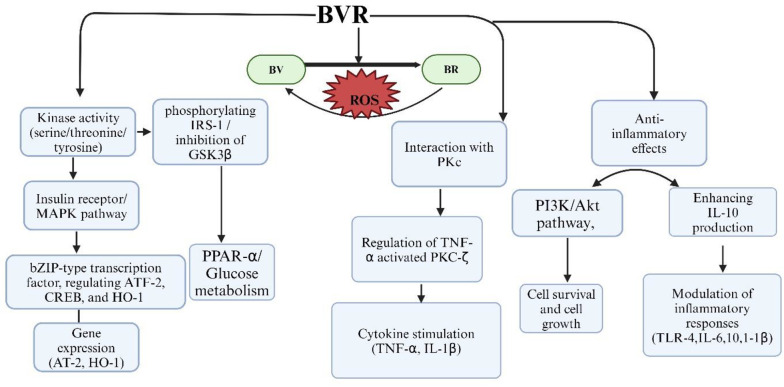
Role of biliverdin reductase (BVR) enzyme with different singalling pathways are illuasted. BVR exhibits kinase activity as a serine/threonine/tyrosine kinase, participating in insulin receptor/mitogen-activated protein kinase (IR-MAPK) pathways and phosphorylating insulin receptor substrate-1 (IRS-1), for glucose metabolism and the inhibition of glycogen synthase kinase-3 beta (GSK-3β). Additionally, BVR functions as an essential leucine zipper domain (bZIP) type transcription factor, regulating transcription factor-2 (ATF-2), cyclic AMP-responsive element-binding protein (CREB) which is essential for gene expression and heme oxygenase-1 (HO-1), which responds to celluar strees. BVR converting biliverdin (BV) to bilirubin (BR), thereby providing antioxidant protection. BVR interacts with protein kinase C (PKC), specifically regulating tumor necrosis factor-alpha (TNF-α) activated PKC-zeta (PKC-ζ) signaling, which stimulate TNF-α and interleukin-1 beta (IL-1β). Furthermore, BVR mediates anti-inflammatory effects through the phosphoinositide-3-kinase and Akt protein kinase (PI3K/Akt) pathway, enhancing interleukine-10 (IL-10) production and modulating the inflammatory response by inhibiting toll-like receptor-4 (TLR-4) expression and affecting cytokines. Created in BioRender. Sharma, R. (2024) https://BioRender.com/t47h170

Moreover, hypoxia-reoxygenation (H-R) injury amplifies ROS production during oxygen bursts, worsening cardiovascular complications and activating various downstream pathways (Figure 1), such as rat sarcoma (RAS), protein kinase C (PKC), mitogen-activated protein kinase (MAPK), extracellular signal-regulated protein kinases 1/2 (ERK1/2), and Janus kinase-signal transducer and activator of transcription (JAK-STAT). These pathways stimulate pro-inflammatory cytokines, including interleukins (IL-1, IL-6) and tumor necrosis factor-alpha (TNF-α) (7, 8). Consequently, oxidative stress and inflammation synergistically enhance the migration and proliferation of vascular smooth muscle cells (VSMCs), leading to vascular remodeling and an increased risk of plaque formation, particularly under conditions of endothelial dysfunction (9).

Among endogenous antioxidants, bilirubin (BR) has emerged as a potent modulator of ROS imbalance. As a product of heme catabolism, BR functions as a powerful lipid-soluble antioxidant (10). Both in vitro and ex vivo studies demonstrate its capacity to prevent oxidant-induced damage and inhibit free radical chain reactions (11). Individuals with mildly elevated unconjugated BR levels, as seen in Gilbert syndrome, exhibit reduced risks of myocardial infarction, heart failure, and ischemic stroke. However, elevated unbound BR levels can lead to kernicterus and liver diseases, including hepatitis and cirrhosis (12).

In contrast, BV has been shown to possess direct antioxidant properties, protecting cells from oxidative stress by scavenging free radicals. It can inhibit NADPH-dependent superoxide production, contributing to its antioxidant and anti-inflammatory effects (13).

However, BV does not match BR in terms of antioxidant capacity, immunomodulatory properties capable of suppressing T-cell responses, benefits in autoimmune conditions, or participation in molecular signaling. Structural differences during the reduction of BV to BR in the presence of BVR result in BV retaining two ketone groups, whereas BR possesses two hydroxyl groups. This structural difference provides BR with an advantage in modulating downstream proteins (14-16).

Indeed, BR is a potent antioxidant; however, its cytoprotective actions are entirely dependent on the availability of BVR. In recent years, BVR has emerged as a dual-specificity kinase, capable of phosphorylating both serine/threonine and tyrosine residues and modulating various downregulating proteins such as MAPK-ERK1/2 signaling (17, 18). Upon activation, ERK1/2 binds to the C and D motifs on BVR, generating a complex known as BVR-ERK1/2. This complex then translocates to the nucleus, phosphorylating several transcription factors, including c-Fos and ELK-1, to promote cell growth and differentiation. Therefore, BVR functions as both an enzymatic regulator for BR and a signaling mediator. These studies suggest that BVR demonstrates multiple cellular functions (17, 19, 20). A depiction of BVR activity and its interaction with various signaling pathways is provided in Figure 1. 

However, in pathophysiological conditions, ERK1/2 binds to the C and D motifs of BVR, diminishing its primary function of reducing BV to BR. This limitation reduces the availability of BR to neutralize ROS, thereby promoting endothelial dysfunction (20).

Animal models with BVR^-/-^ have been employed to study the activity of BV and BR (21). However, while a few drugs influence BVR activity, there is currently no direct inhibitor that specifically targets BVR or its gene expression (22). This absence of a targeted inhibitor restricts the comprehensive assessment of BVR's role as a dual-specificity kinase enzyme, thereby limiting deeper exploration of its regulatory functions and therapeutic potential. 

## 2. Objectives

In this study, we investigated the role of BVR in ERK1/2 signaling by inhibiting ERK1/2 with PD-98059. We evaluated the effects of BR, the oxidant 22'-Azobis (2-amidinopropane) dihydrochloride (ABAP), and the ERK1/2 inhibitor PD-98059 on the relaxation and maximal effect (Emax) of aortic rings under normoxic and H-R conditions. This approach highlights BVR’s therapeutic potential as a target for cardiovascular diseases. 

## 3. Methods and Results

### 3.1. Chemical Used

Bilirubin and ABAP were obtained from Sigma Aldrich, Switzerland, while dimethyl sulfoxide (DMSO) was sourced from Merck, Germany.

Fresh rat hearts were rinsed to remove blood and immediately placed in a container filled with cold (4°C) Krebs-Henseleit (K-H) solution of the following composition: Potassium chloride (KCl) 4.7 × 10^-3^ M, CaCl_2_ 2.5 × 10^-3^ M, NaHCO_3_ 2.5 × 10^-2^ M, MgSO_4_ 1.2 × 10^-3^ M, KH_2_PO_4_ 1.2 × 10^-3^ M, and glucose 1.1 × 10^-2^ M (all from Merck, Darmstadt, Germany). Aortic rings were cleaned of fat and connective tissue and cut into rings (3 - 4 mm in length).

After washing, the aortic rings were immediately mounted in EMKA organ baths filled with K-H solution, maintained at 37°C, and continuously bubbled with a 95% O_2_ - 5% CO_2_ mixture (Messer Ruše, Slovenia). Once mounted, the rings were allowed to equilibrate at a 5.0 g resting tension for 60 minutes, followed by three contractions with 6.0 × 10^-2^ M KCl to achieve stable contractions.

Initially, the aortic rings were stabilized in an organ bath containing physiological saline solution at 37°C for 1 hour. Subsequently, the rings were treated with acetylcholine (ACh) at concentrations ranging from 10^-8^ to 10^-5^ M to assess endothelium-dependent relaxation. Phenylephrine (PE) was then applied at similar concentrations to induce contraction and assess vascular reactivity.

For H-R experiments, the rings were exposed to hypoxia (95% N_2_, 5% CO_2_) at 37°C for 3 hours to simulate hypoxic conditions. Following hypoxia, the rings were allowed to relax for 1 hour to stabilize before reoxygenation. Reoxygenation was performed using a 95% O_2_ - 5% CO_2_ mixture at 37°C for 3 hours to simulate reperfusion. 

**Figure 2.**
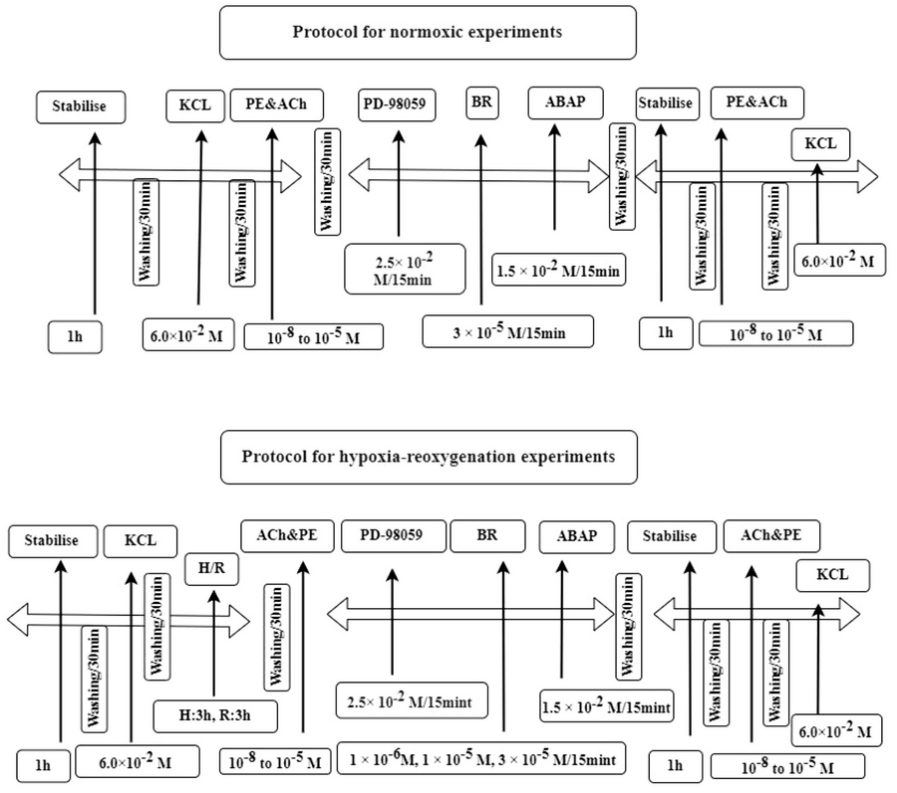
Outlines of normoxic and hypoxia-reoxygenation (H-R) experimental protocols. The normoxic protocol involved a series of steps including stabilisation, the addition of potassium chloride (KCl), acetylcholine and phenylephrine (ACh & PE), the inhibitor PD-98059 and bilirubin (BR) and 22'-Azobis (2-amidinopropane) dihydrochloride (ABAP). This sequence was repeated with another stabilisation phase and reintroduction of Ach, PE and KCl. The second protocol was for H-R experiments and followed a similar sequence but included an additional H-R phase, where H for hypoxia and R for reoxygenation.

After reoxygenation, the rings were treated with various drugs, including BR, ABAP, and PD-98059, to evaluate their effects. Subsequently, ACh and PE were reintroduced at the same concentrations to assess changes in relaxation. The experimental protocols for both normoxic and H-R conditions are illustrated in Figure 2. 

### 3.2. Data Analysis

The data were presented as mean ± standard error of the mean (SEM), where (n) represents the number of arterial rings and (N) represents the number of animals. To evaluate statistical significance, one-way and two-way analysis of variance (ANOVA) were performed, followed by Bonferroni’s post-test for inter-comparisons, using GraphPad Prism 8.0 software. A P-value of less than 0.05 was considered statistically significant.

### 3.3. Effect of Bilirubin Under Normoxic Conditions

**Figure 3.**
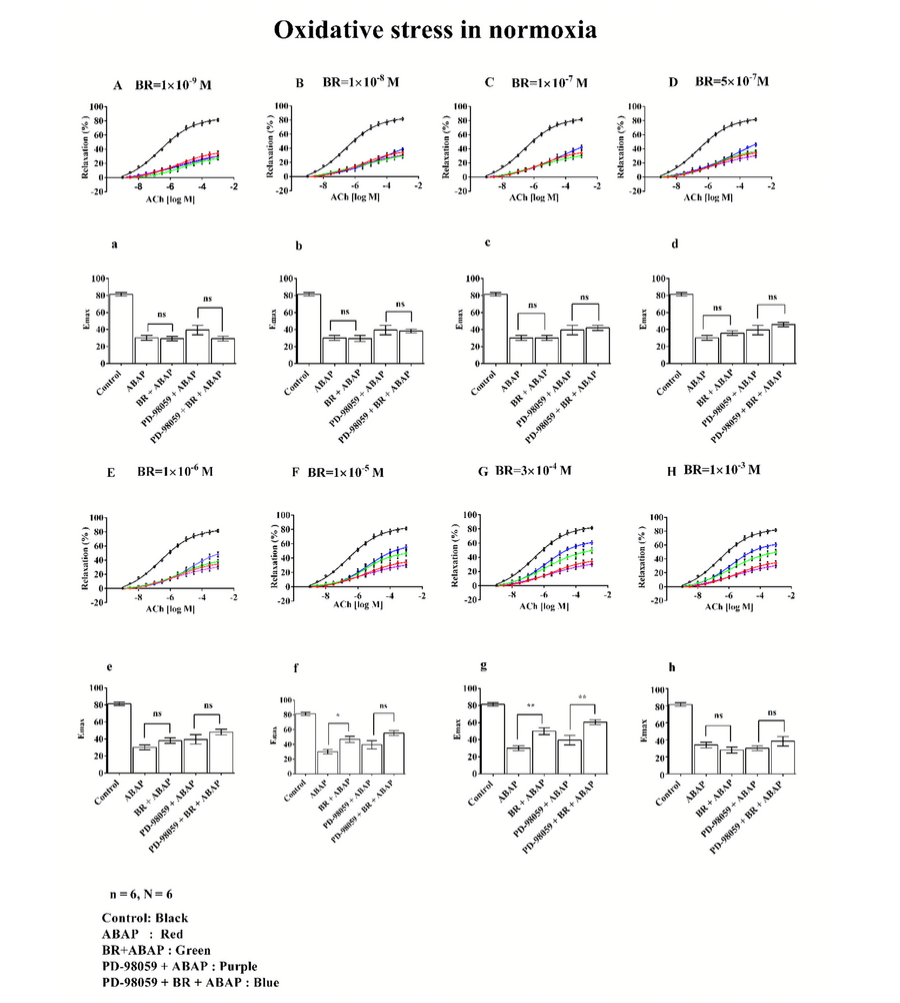
Effects of different concentrations of bilirubin (BR) with 22'-Azobis (2-amidinopropane) dihydrochloride (ABAP) and PD-98059 on concentration-response curves (A to H) and maximal effect (Emax) (a to h) on aortic rings under oxidative stress. Where, (n) represented the number of arterial rings, and (N) represented the number of animals, with 6 rings per group, each ring from a different animal (N = 6). Data were presented as mean ± standard error of the mean (SEM). Statistical significance was determined using one-way analysis of variance (ANOVA) with post-hoc Bonferroni test, where ns: Non-significant; *: P ≤ 0.05 indicated significance; and **: P ≤ 0.01 indicated high significance.

The effects of different concentrations of BR on relaxation and Emax in rat aortic rings are presented in Figure 3. Graphs A/a (1 × 10^-9^ M), B/b (1 × 10^-8^ M), C/c (1 × 10^-7^ M), D/d (5 × 10^-7^ M), E/e (1 × 10^-6^ M), F/f (1 × 10^-5^ M), and G/g (3 × 10^-4^ M), along with H/h (1 × 10^-3^ M), illustrate the dose-dependent changes observed under normoxic conditions.

The control group demonstrated approximately 80% relaxation and Emax. In contrast, treatment with ABAP resulted in a marked decrease, showing around 30% relaxation and Emax. In Graphs A/a to E/e, depicting the effects of BR (bilirubin) at concentrations ranging from 1 × 10^-9^ M to 1 × 10^-6^ M, no notable changes in % relaxation or Emax were observed, even when combined with PD-98059.

However, at a BR concentration of 1 × 10^-5^ M (Graph F/f), there was a significant increase in both relaxation and Emax in the BR + ABAP group (exceeding 50%) compared to ABAP alone.

Similarly, BR at 3 × 10^-4^ M (Graph G/g) showed notable relaxation and Emax (approximately 60%) relative to ABAP alone. A significant difference was also observed in the PD-98059 + BR + ABAP group compared to PD-98059 + ABAP alone.

At a BR concentration of 1 × 10^-3^ M (Graph H/h), no changes in relaxation or Emax were observed.

### 3.4. Effect of Bilirubin Under Hypoxia-Reoxygenation Injury

**Figure 4.**
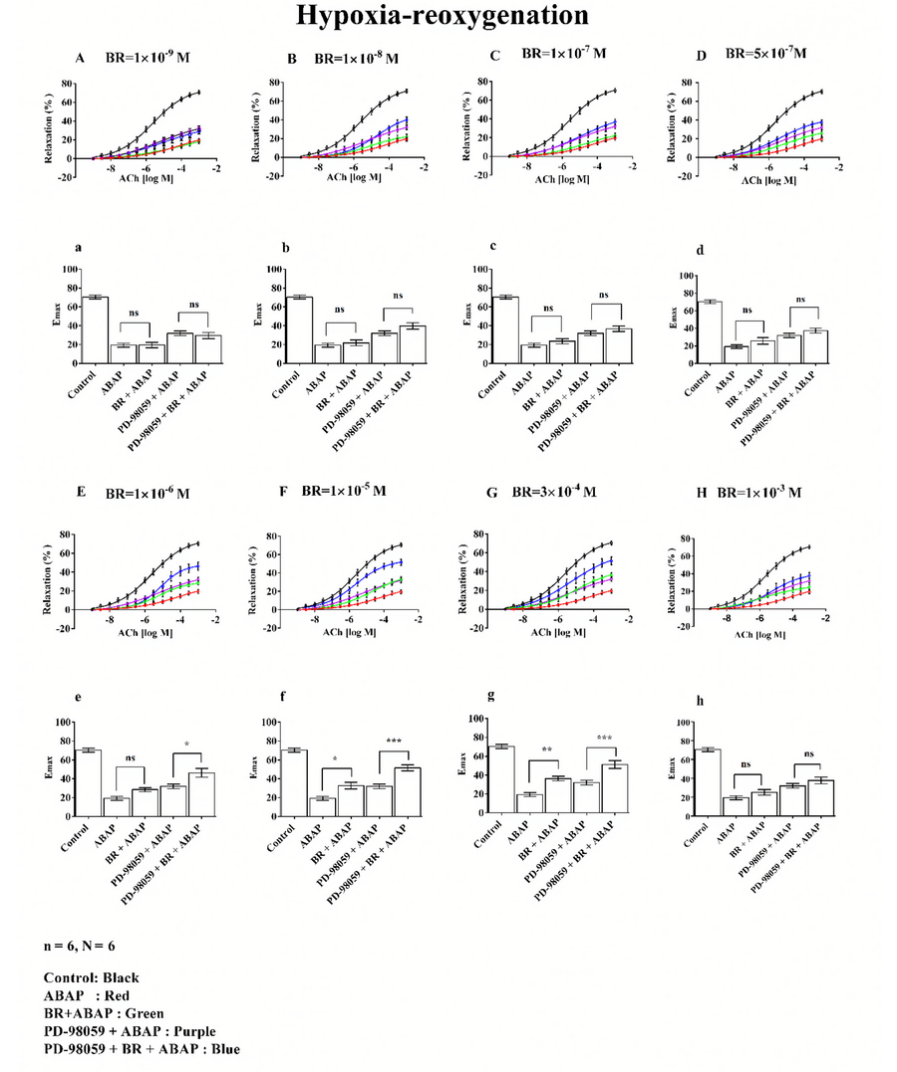
The effects of different concentrations of bilirubin (BR) with 22'-Azobis (2-amidinopropane) dihydrochloride (ABAP) and PD-98059 on concentration-response curves (A to G) and Emax (a to g) on aortic rings under hypoxia-reoxygenation (H-R) were studied. Where, (n) indicated the number of arterial rings and (N) indicated the number of animals with 6 rings per group each ring from a different animal (N = 6). Data were presented as mean ± standard error of the mean (SEM). Statistical significance was determined using a one-way analysis of variance (ANOVA) with posthoc Bonferroni test, where ns: Non-significant; *: P ≤ 0.05; **: P ≤ 0.01; ***: P ≤ 0.001 indicated significance indicated significance.

Relaxation and Emax at seven different concentrations of BR in the presence of ABAP and PD-98059 under H-R conditions are shown in Figure 4. The figure is divided into two sections: A to H represent relaxation curves, and a to g depict Emax.

Graphs A/a (1 × 10^-9^ M), B/b (1 × 10^-8^ M), C/c (1 × 10^-7^ M), D/d (5 × 10^-7^ M), E/e (1 × 10^-6^ M), F/f (1 × 10^-5^ M), and G/g (3 × 10^-4^ M) illustrate the dose-dependent changes evaluated under H-R conditions.

The control group demonstrated approximately 70% relaxation and Emax, while ABAP treatment reduced these values to around 20%. Graphs A/a to D/d, representing BR concentrations from 1 × 10^-9^ M to 5 × 10^-7^ M, showed no significant changes in % relaxation or Emax, even when PD-98059 was added.

At a BR concentration of 1 × 10^-6^ M (Graph E/e), a significant increase in relaxation and Emax was observed, reaching approximately 50% in the PD-98059 + BR + ABAP group compared to about 30% in the PD-98059 + ABAP group. Similarly, at 1 × 10^-5^ M (Graph F/f), BR exhibited enhanced relaxation and Emax, with values around 40% in the PD-98059 + BR + ABAP group compared to 30% in the PD-98059 + ABAP group. At 3 × 10^-4^ M (Graph G/g), this effect was further amplified, with relaxation and Emax exceeding 50% relative to ABAP alone.

In contrast, at a BR concentration of 1 × 10^-3^ M (Graph H/h), BR did not produce significant changes in relaxation or Emax when comparing ABAP alone with BR + ABAP or PD-98059 + ABAP with PD-98059 + BR + ABAP.

## 4. Discussion

The study investigated the protective role of BR and ERK1/2 inhibition in mitigating oxidative stress and H-R injury in the rat aorta. Aortic rings were treated with varying concentrations of BR, which effectively countered the negative impact of ROS induced by ABAP under both normoxic conditions and H-R injury. Reactive oxygen species generated by ABAP further activated the ERK1/2 signaling pathway, with ERK1/2 inhibition observed in the combination of PD-98059 + BR. Notably, at higher concentrations (1 × 10^-3^ M), the BR did not exhibit protective effects, while its efficacy was more pronounced at lower concentrations.

**Figure 5.**
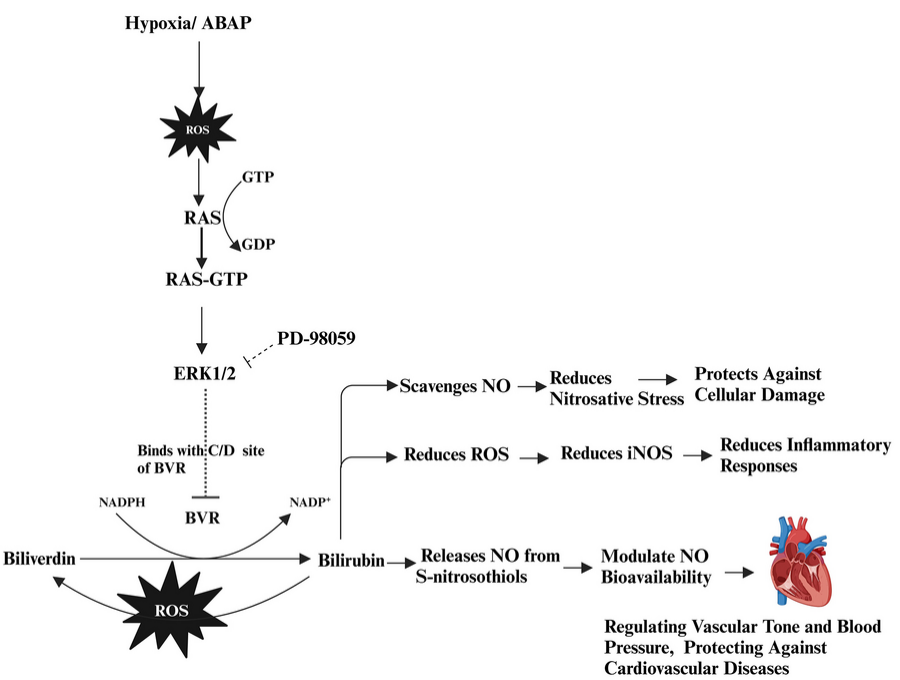
Oxidative stress via reactive oxygen species (ROS)/hypoxia- reoxgenation (H-R) activates signaling pathways, leading to various cellular responses. Pro-oxidant 22'-Azobis (2-amidinopropane) dihydrochloride (ABAP) along with H-R injury increase ROS, triggering the rat sarcoma (RAS)-GTP activates extracellular signal-regulated protein kinases 1/2 (ERK1/2) that binds with C and D motifs of biliverdin reductase (BVR) enzyme and reduces the reducation of biliverdin (BV) to bilirubin (BR). BR modulates nitric oxide (NO) bioavailability, protecting against cellular damage, inflammation, and cardiovascular diseases. Where as PD-98059, inhibits the ERK1/2 therefore, BVR works on bile pigments. Created in BioRender. Sharma, R. (2024) https://BioRender.com/i71j451

The ERK1/2 signaling pathway, which is essential for numerous physiological processes, has been implicated in pathological conditions through its role in promoting excessive ROS in cardiovascular dysfunctions (23). Furthermore, ERK1/2 inhibition via PD-98059 enhanced BR’s antioxidant protective function, suggesting that ERK1/2 activation may hinder BR’s effectiveness in mitigating oxidative stress. The combination of BR and PD-98059 was particularly effective in disrupting the BVR/ERK1/2 axis, resulting in improved vascular relaxation and Emax compared to either treatment alone. The interaction between BVR and ERK1/2 is illustrated in Figure 5. 

Our research aligns with previous studies demonstrating that BR exhibits antioxidant effects by neutralizing ROS and protecting endothelial functions. However, in the absence of BVR, the antioxidant actions of BR are significantly diminished (24). Inhibiting ERK1/2 while enhancing 

BVR activity enhances the production of BR, offering protection against ROS, a major contributor to cardiovascular diseases. The BVR functions as a dual-specificity kinase (Ser/Thr and Tyr), playing a crucial upstream role in the IGF-1 and MAPK signaling pathways. It regulates key signaling molecules, such as PKC and ERK1/2, which are involved in endothelial dysfunction, inflammation, and cell survival. This pathway is critical in oxidative stress as it promotes cell survival by limiting ROS generation (25).

Our study demonstrated that inhibiting ERK1/2 with PD-98059 may prevent the binding of ERK1/2 to the C and D binding sites of BVR. This allows BVR to remain available for converting BV to BR. Disrupting the BVR/ERK1/2 axis may interfere with the PI3K/Akt pathway, potentially increasing apoptosis and exacerbating ROS levels.

While earlier studies have explored the protective effects of BR, limited research has focused on how the ERK1/2 pathway modulates BVR activity to enhance BR's protective functions. Our findings address this gap by showing that inhibiting ERK1/2 with PD-98059 significantly improves BVR activity, leading to increased BR production and enhanced ROS protection. The synergistic interaction between BR and the ERK1/2 inhibitor PD-98059 highlights the therapeutic potential of this combination. 

In conclusion, these findings underscore the potential therapeutic value of targeting the BVR/ERK1/2 signaling axis. Inhibiting ERK1/2 may serve as a promising strategy to enhance BVR's antioxidant role, offering new avenues for the treatment of cardiovascular diseases.

## Data Availability

The dataset presented in the study is available on request from the corresponding author during submission or after publication. The data are not publicly available due to our research involves the systematic screening of drugs that modulate BVR activity at varying concentrations. Consequently, it is not feasible to publish the data publicly.
